# A Salivary Clear Cell Tumor With an Unclear Diagnosis: A Report of a Rare Case

**DOI:** 10.7759/cureus.73656

**Published:** 2024-11-14

**Authors:** Svyat Strokov, Raybaud Helène, Nathalie Cardot-Leccia, Bérangère Dadone-Montaudié, Christine Voha

**Affiliations:** 1 Oral Pathology, Faculté de Chirurgie Dentaire, Université Côte d'Azur, Nice, FRA; 2 Oral Pathology, Institut de Médecine Bucco-Dentaire, Centre Hospitalier Universitaire (CHU) de Nice, Nice, FRA; 3 Oral Medicine, Faculté de Chirurgie Dentaire, Université Côte d'Azur, Nice, FRA; 4 Dermatopathology, Laboratoire Central d'Anatomo-Pathologie de Nice, Pôle Oncologie Moléculaire, Nice, FRA; 5 Oncology, Laboratoire Central d'Anatomo-Pathologie de Nice, Pôle Oncologie Moléculaire, Nice, FRA; 6 Dentistry, Institut de Médecine Bucco-Dentaire, Centre Hospitalier Universitaire (CHU) de Nice, Nice, FRA; 7 Dentistry, Université Côte d'Azur, Nice, FRA

**Keywords:** clear cells, malignant salivary gland tumor, minor salivary gland, oral cavity, salivary gland tumor

## Abstract

The histopathological diagnosis of salivary tumors is considered complex, due to their histological, phenotypic, and genotypic diversity. There are numerous tumors with morphological and/or immunohistochemical aspects that are superimposable but require very different treatment. In this context, salivary lesions containing clear cells are numerous and form part of the diagnostic challenges. We present the case of a 63-year-old woman with a tumor of the accessory salivary glands of the palate, with a predominant clear cell contingent. The aim of this paper is to report a case of a clear cell salivary tumor and to detail the difficulties of differential diagnosis, highlighting new data in the literature and the role of molecular biology.

## Introduction

Salivary gland tumors are rare, accounting for 3-6% of all head and neck tumors, with an incidence ranging from 0.4 to 13.5 per 100,000 cases, depending on the study [1,2]. In 2022, the World Health Organization (WHO) published its fifth classification of head and neck tumors, including 36 salivary tumors, with 15 benign and 21 malignant lesions [3]. Fortunately, benign tumors are the most common in 65% of cases, with pleomorphic adenoma and Warthin's tumor being the most common. In 35% of cases, the tumors are malignant, and the most common subtypes are mucoepidermoid carcinoma, adenoid cystic carcinoma, and polymorphic adenocarcinoma [4]. The parotid gland is the most commonly affected site (50-60% of cases), followed by the accessory salivary glands, the submandibular gland, and, less commonly, the sublingual gland [1,4]. Curiously, the smaller the gland, the greater the risk of malignancy: tumors arising from the accessory salivary glands or the submandibular gland are more often malignant [1]. Clinically, these tumors usually cause more or less symptomatic, nonspecific swelling. Their histologic diagnosis is considered complex due to their heterogeneity and the overlap of their histologic or immunohistochemical appearance [3]. Classifications of salivary tumors are evolving rapidly, with the discovery of new entities due to advances in molecular biology, which is increasingly allowing the characterization of the profile of molecular alterations in these tumors [5]. The aim of this work is to present the diagnostic approach in the case of a complex salivary tumor with a predominance of clear cells and to detail the differential diagnosis using immunohistochemical and genetic data.

## Case presentation

**Figure 1:**
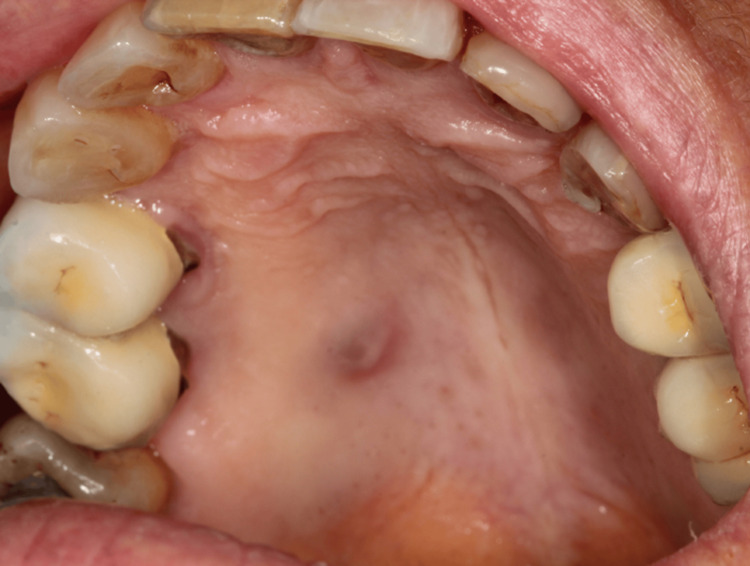
Clinical photograph showing a bluish nodule 5-6 mm in diameter, sessile, located on the right side of the hard palate

We present the case of a 63-year-old woman in good general health who presented with an asymptomatic nodule on the hard palate. Clinical examination revealed a 5-6 mm bluish nodule on the hard palate (Figure 1).

**Figure 2:**
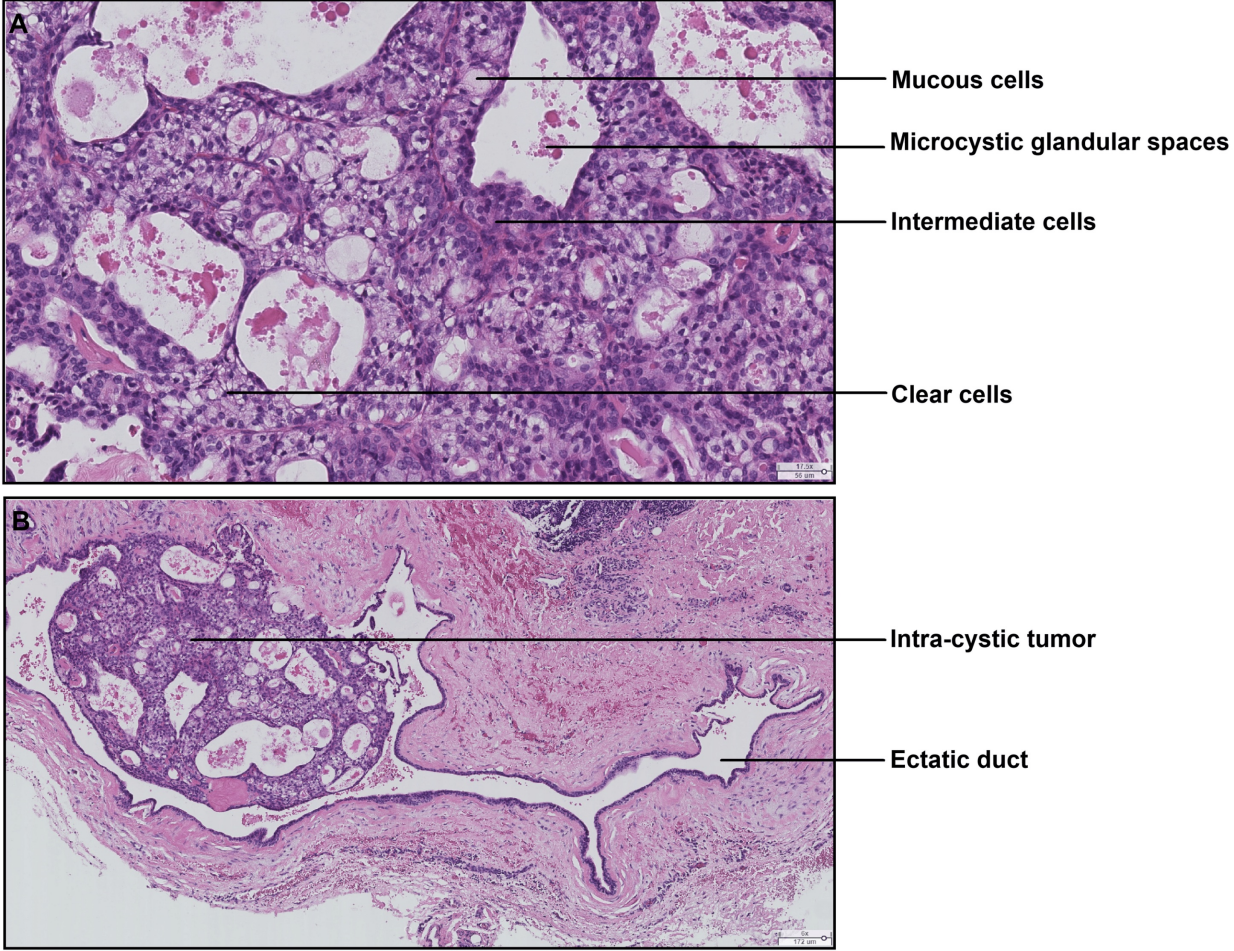
Histological section showing an intra-cystic tumoral proliferation (A) Microphotograph (hematoxylin-eosin stain, original magnification ×175) showing a tumor proliferation hollowed out by glandular microcystic spaces and bordered by clear cells, a few intermediate cells, and very rare mucus cells (legends). (B) Microphotograph (hematoxylin-eosin stain, original magnification ×60) showing in situ tumor proliferation developed at the expense of an ectatic salivary duct.

Palpation of the lymph nodes did not reveal any suspicious nodes. The clinical differential diagnosis includes benign or malignant tumors of the salivary glands or a non-neoplastic process such as a mucocele. A biopsy was performed under local anesthesia. Histologic examination revealed two well-defined nodules with glandular and cystic structures developed at the expense of a salivary duct (Figure 2A, 2B).

**Figure 3:**
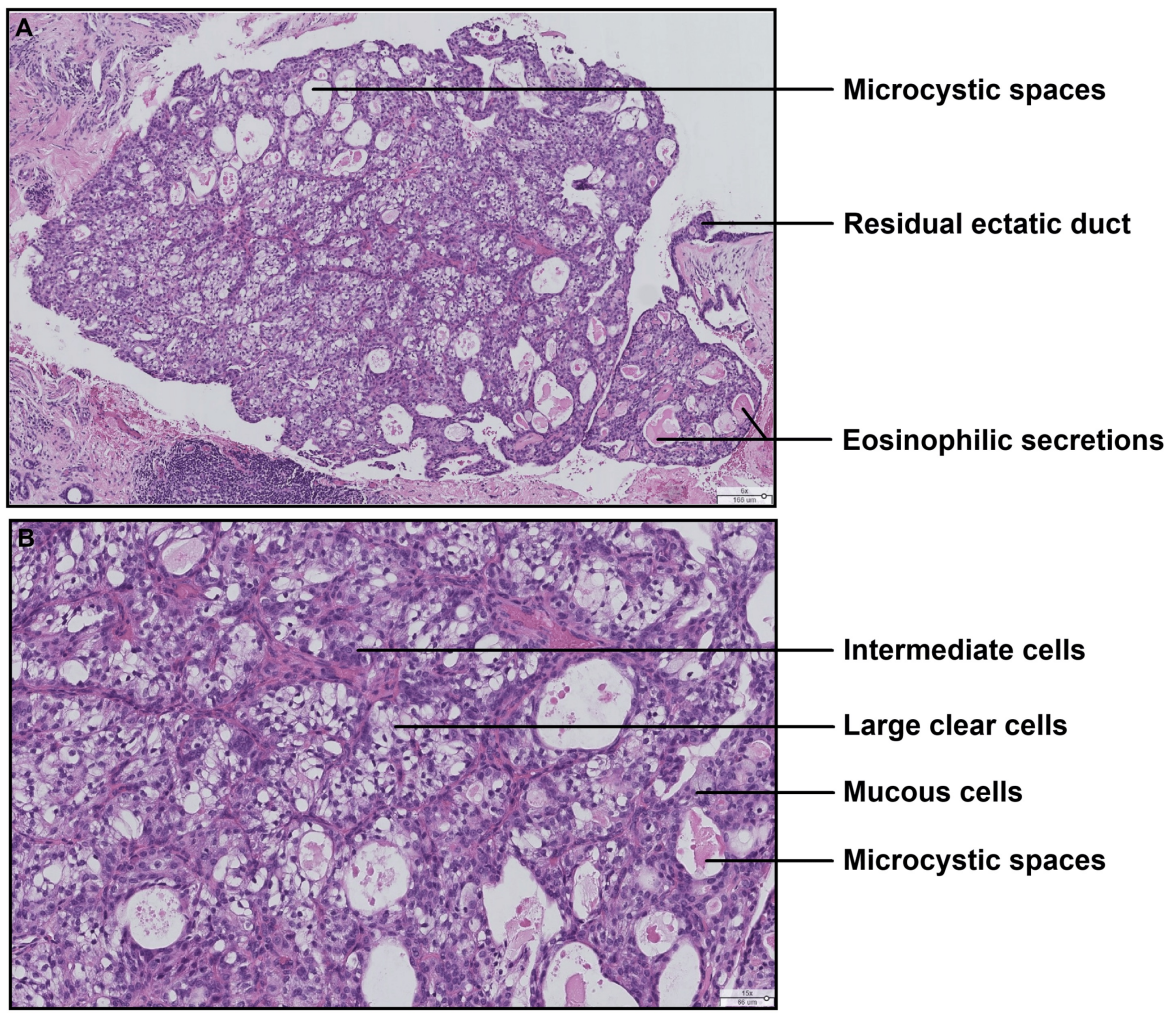
Histological section showing the three cellular contingents of the tumor (A) Microphotograph (hematoxylin-eosin stain, original magnification ×60) showing a tumoral nodule with microcystic spaces, sometimes filled by an eosinophilic pseudocolloidal secretory substance. (B) Microphotograph (hematoxylin-eosin stain, original magnification ×150) showing the morphology of the cellular contingents with a majority population of cells with large clear cytoplasm, intermediate cells with amphophilic cytoplasm and rounded nuclei, and very rare mucous cells.

These nodules are composed of predominantly clear cells, cells with rounded nuclei and eosinophilic cytoplasm, and very rare mucous cells (Figure 3A, 3B). No invasive territory was observed within the borders of the specimen: the tumor developed only in one salivary duct. No cytonuclear atypia, mitoses, intravascular tumor emboli, or neurotropism were observed.

Faced with a muco-secreting lesion with cystic architecture and clear cellularity, several differential diagnoses are evoked. The spectrum of malignant lesions includes clear cell mucoepidermoid carcinoma, clear cell hyalinized carcinoma, secretory carcinoma, epithelial-myoepithelial carcinoma, myoepithelial carcinoma, and, in principle, metastasis of clear cell renal cell carcinoma. Among the benign lesions, clear cell oncocytoma, clear cell myoepithelioma, mucinous cystadenoma, and clear cell intraductal papilloma must be excluded.

**Figure 4:**
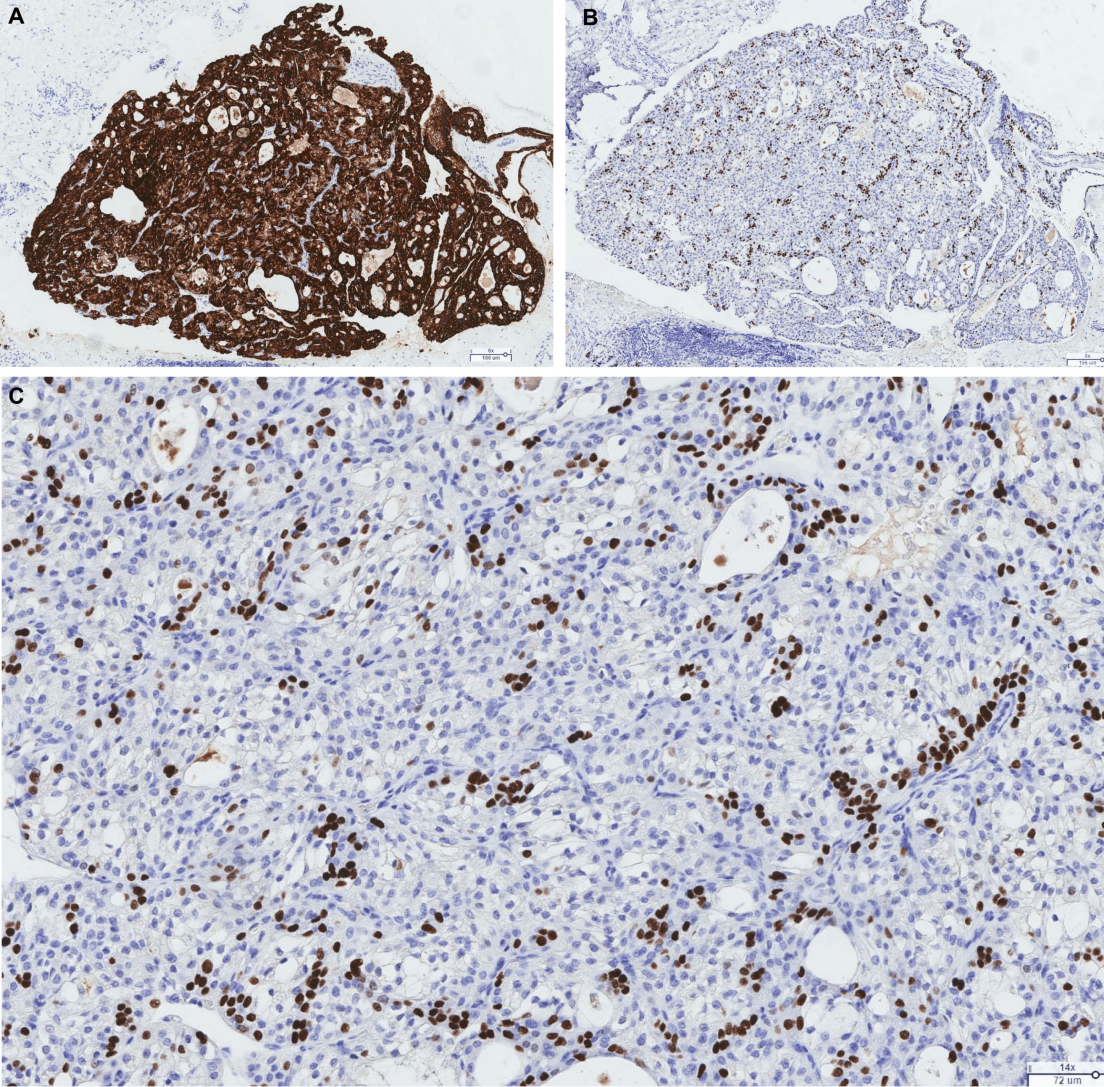
Positive immunohistochemical markers (A) CK7 (original magnification ×60): homogeneous and diffuse cytoplasmic positivity within the tumor, suggestive of salivary differentiation. (B) p63 (original magnification ×60): heterogeneous nuclear positivity within the tumor. (C) p63 (original magnification ×140): heterogeneous nuclear positivity, rather peripheral but indicative of basal differentiation.

**Figure 5:**
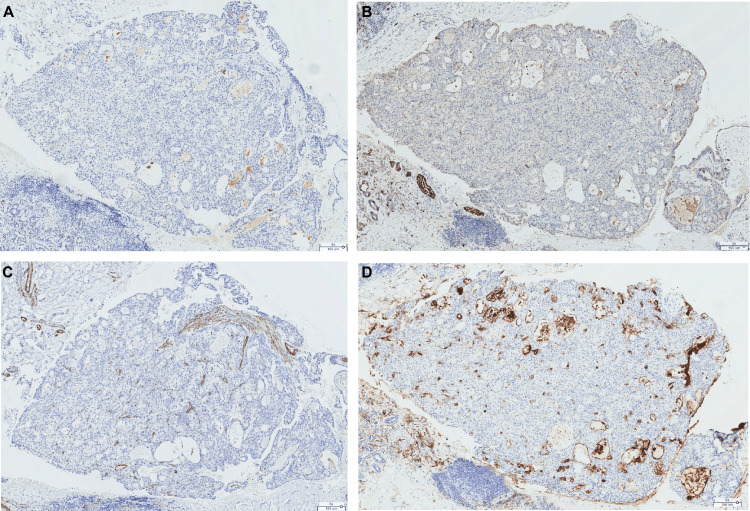
Negative immunohistochemical staining (A) Antibody anti-SOX-10, negative staining (original magnification ×60), indicating the absence of pure acinar differentiation. (B) Antibody anti-S-100 protein, negative staining (original magnification ×60), indicating the absence of myoepithelial differentiation and ruling out secretory carcinoma. (C) Antibody anti-smooth muscle actin, negative staining (original magnification ×60), indicating the absence of myoepithelial differentiation and ruling out biphasic or pure myoepithelial tumors (epithelial-myoepithelial carcinoma, myoepithelial carcinoma, etc.). (D) Antibody anti-CD10, negative staining (original magnification ×60), an argument to rule out metastasis of clear cell renal cell carcinoma.

Immunohistochemically, the tumor expressed CK7 and p63 (peripherally) (Figure 4A, 4B, 4C). It was negative for smooth muscle actin (SMA), SOX-10, S-100 protein (PS-100), and CD10 (Figure 5A, 5B, 5C, 5D).

Negativity for actin excludes tumors with a myoepithelial component, such as epithelial-myoepithelial carcinoma and myoepithelial carcinoma. S-100 protein negativity rules out secretory carcinoma. CD10 negativity excludes metastatic renal cell carcinoma.

The absence of hyalinized stroma does not support the diagnosis of hyalinized clear cell carcinoma. The absence of papillary architecture rules out intraductal papilloma. The presence of intermediate cells rules out clear cell oncocytoma, which has a much more monotonous morphology.

The final diagnosis is a clear cell variant of intra-cystic (in situ) mucoepidermoid carcinoma. This diagnosis was formally confirmed by a CRTC1-MAML2 fusion found in molecular biology.

## Discussion

Clear cells are found in 1-2% of head and neck lesions. The clear cell appearance is due to an intracytoplasmic accumulation of glycogen, water, mucins, and lipids or can be explained by artifactual phenomena [6,7]. These clear cell tumors may be of salivary origin or, less commonly, of odontogenic, mesenchymal, or, exceptionally, metastatic origin [7]. When they represent the majority of tumors, the diagnostic challenge can be considerable because many tumors share the same morphologic features and the same tumor can have several variants with very different histologic presentations [8]. Moreover, the differential diagnosis between benign and malignant lesions can be very difficult because these clear cell tumors tend to be low-grade and generally contain few cytonuclear atypia [6]. The diagnostic approach must therefore be rigorous, based on a strict correlation of histological, immunohistochemical, and genetic aspects [3].

Considering an oral tumor with clear cell change, metastatic origin must first be excluded by comparing the clinical history with an immunohistochemical study. Anti-CD10 staining should always be performed to exclude metastasis from clear cell renal cell carcinoma [9].

If the salivary origin is confirmed by immunohistochemistry, the tumor should be classified as myoepithelial, with labeling directed against calponin and/or smooth muscle actin [10]. Some tumors are purely epithelial (such as mucoepidermoid carcinoma or hyalinized clear cell carcinoma), while others are biphasic (such as epithelial-myoepithelial carcinoma) or purely myoepithelial (myoepithelial carcinoma).

In our case, the differential diagnosis consisted mainly of salivary tumors with a clear cell component (Table 1 and Table 2).

**Table 1:** Differential diagnosis of malignant clear cell salivary tumors SMA: smooth muscle actin References: [1] and [3]

Differential diagnosis	Morphology	Immunoprofile	Molecular profiling	Clinical prognosis
Mucoepidermoid carcinoma (clear cell variant)	Mucous, intermediate, and squamoid cells with a variable amount of clear cells	Positive for basal markers (p63/p40). Negative for myoepithelial markers (SMA, calponin, etc.). Negative for SOX10/PS-100	MAML2 rearrangement (CRTC1/3::MAML2)	Five-year overall survival rates of 90% for low-grade tumors and 55% for high-grade tumors
Secretory carcinoma	Microcystic, tubular, or solid pattern with abundant eosinophilic "colloid-like" secretions	Negative for basal markers (p63/p40). Negative for myoepithelial markers (SMA, calponin, etc.). Positive for S100 and mammaglobin	ETV6::NTRK3 fusion	Good overall, indolent tumor (mean disease-free survival: 12 years)
Hyalinizing clear cell carcinoma	Infiltrative sheets of monotonous clear cells in a dense hyalinized stroma. Perineural invasion is frequent	Positive for basal markers (p63/p40). Negative for myoepithelial markers (SMA, calponin, etc.)	EWSR1::ATF1 fusion	Excellent prognosis; death of disease is rare
Epithelial-myoepithelial carcinoma	Bilayered tubule with cuboidal ductal cells surrounded by outer clear myoepithelial cells	Positive for both epithelial and myoepithelial markers. Positive for SOX-10 and S100 protein	HRAS mutation	Usually good prognosis (five-year survival: 91%)
Myoepithelial carcinoma	Invasive growth; myoepithelial cells with epithelioid, cleared, spindle, or plasmacytoid morphology. Absence of ducts	Positive for basal markers (p63/p40). Positive for myoepithelial markers (SMA, calponin, etc.)	PLAG1 mutation commonly seen, especially in myoepithelial carcinoma, e.g., pleomorphic adenoma, sometimes EWSR1 rearrangement	Relatively aggressive; five-year survival rate: 65%; recurrences are common

**Table 2:** Differential diagnosis of benign clear cell salivary tumors References: [1] and [3]

Differential diagnosis	Morphology	Immunoprofile	Molecular profiling	Clinical prognosis
Clear cell oncocytoma	Large polygonal oncocytic or cleared cells	Basal markers: positive. Myoepithelial markers: positive. SOX-10/PS-100 protein: negative	-	Recurrences are rare
Clear cell variant of myoepithelioma	Encapsulated tumor composed entirely of myoepithelial cells with spindle, epithelioid, clear, or plasmacytoid morphology	Basal markers: positive. Myoepithelial markers: positive. SOX-10/S-100 protein: positive	PLAG1 sometimes	Low recurrence
Salivary cystadenoma	Multicystic spaces with intraluminal papillary projections with cuboidal, mucous, or oncocytic cells	Basal markers: positive (basal cell layer). SOX-10/S-100 protein: negative	-	Recurrences are rare
Intraductal papilloma	Unicystic cavity filled with papillary projections (columnar cells with rare mucoid cells)	Little help in diagnosis	-	Recurrences are rare

We prioritized the differential diagnosis by considering firstly the clear cell variant of mucoepidermoid carcinoma, due to the presence of clear cells and cells with rounded nuclei showing "intermediate" morphology; secondly, hyalinized clear cell carcinoma, due to the predominance of these clear cells in our case; and, finally, secretory carcinoma, due to the presence of glandular spaces filled with pseudocolloidal secretory material.

Immunohistochemistry allows us to exclude other entities with a myoepithelial component, as well as secretory carcinoma, which is always positive for PS-100 and mammaglobin [3]. On the other hand, immunohistochemical aspects are superimposable between hyalinized clear cell carcinoma and mucoepidermoid carcinoma. Moreover, hyalinized clear cell carcinoma can sometimes contain both mucinous cells and squamous cells, making differential diagnosis almost impossible [1,3,11]. In our case, only the CRTC1::MAML2 fusion found in molecular biology allowed us to make the differential diagnosis and conclude that it was a mucoepidermoid carcinoma.

Mucoepidermoid carcinoma is the most common primary malignant salivary tumor according to most incidence studies [4]. It accounts for 10% of all salivary tumors and 25% of all malignant tumors and can affect individuals of any age, including children, with no gender preference [12].

In 60-80% of cases, this tumor is associated with a specific genetic fusion CRTC1 or CRCT3::MAML2 [8], which provides a molecular signature for diagnosis, but also has prognostic value, as MAML2-positive tumors are associated with a much better survival rate [13].

Conventionally, mucoepidermoid carcinomas are defined by a triple epidermoid, mucinous, and intermediate content. These morphologic aspects are an important diagnostic argument for this entity, even in the absence of invasion [14]. However, a recent review by Bishop et al. 2022 shows that this definition needs to be reconsidered, as these tumors may lack the squamous component (p63+, p40+) [15]. In our case, this contingent was very discrete, with focal and peripheral positivity for p63, making the diagnosis of mucoepidermoid carcinoma less certain. Our case thus illustrates the value of molecular biology, which can sometimes be used to establish the diagnosis of certain tumors with atypical histologic presentations.

## Conclusions

The histological diagnosis of clear cell tumors of the head and neck is complex because these cells are found in a wide variety of entities, benign or malignant, of salivary, epithelial, odontogenic, or sometimes metastatic origin. This diagnostic difficulty is illustrated by this clinical case, which presents a salivary tumor without histological criteria of malignancy or invasion and without epidermoid contingent, but which could be subtyped as a mucoepidermoid carcinoma, thanks to its molecular profile. Thus, genotyping techniques are sometimes necessary when clinical, histological, and immunohistochemical comparisons are not sufficient to make a definitive diagnosis.
